# Differences in Steady-State Erythropoiesis in Different Mouse Bones and Postnatal Spleen

**DOI:** 10.3389/fcell.2021.646646

**Published:** 2021-05-13

**Authors:** Vamsee D. Myneni, Ildikó Szalayova, Eva Mezey

**Affiliations:** Adult Stem Cell Section, Craniofacial and Skeletal Diseases Branch, National Institute of Dental and Craniofacial Research (NIDCR), National Institutes of Health (NIH), Bethesda, MD, United States

**Keywords:** erythropoiesis, erythroid progenitors, erythroid precursors, different mouse bones, cortical and trabecular bone

## Abstract

Adult erythropoiesis is a highly controlled sequential differentiation of hematopoietic stem cells (HSCs) to mature red blood cells in the bone marrow (BM). The bones which contain BM are diverse in their structure, embryonic origin, and mode of ossification. This has created substantial heterogeneity in HSCs function in BM of different bones, however, it is not known if this heterogeneity influences erythropoiesis in different bones and different regions of the same bone. In this study, we examined steady state BM erythroid progenitors and precursors from different bones – the femur, tibia, pelvis, sternum, vertebrae, radius, humerus, frontal, parietal bone, and compared all to the femur. Trabecular and cortical regions of the femur were also compared for differences in erythropoiesis. In addition, mouse spleen was studied to determine at which age erythropoietic support by the spleen was lost postnatally. We report that total erythroid cells, and erythroid precursors in the femur are comparable to tibia, pelvis, humerus and sternum, but are significantly reduced in the vertebrae, radius, frontal, and parietal bones. Erythroid progenitors and multipotential progenitor numbers are comparable in all the bones except for reduced number in the parietal bone. In the femur, the epiphysis and metaphysis have significantly reduced number of erythroid precursors and progenitors, multipotential progenitors and myeloid progenitors compared to the diaphysis region. These results show that analysis of erythroid precursors from diaphysis region of the femur is representative of tibia, pelvis, humerus and sternum and have significant implications on the interpretation of the steady-state erythropoiesis finding from adult BM. Postnatal spleen supports erythroid precursors until 6 weeks of age which coincides with reduced number of red pulp macrophages. The residual erythroid progenitor support reaches the adult level by 3 months of age. In conclusion, our findings provide insights to the differences in erythropoiesis between different bones, between trabecular and cortical regions of the femur, and developmental changes in postnatal spleen erythropoiesis.

## Introduction

Adult erythropoiesis is a highly controlled sequential differentiation of hematopoietic stem cells (HSCs) into mature red blood cells (RBCs) in the bone marrow (BM). At steady state, adult humans make more than 2.5 × 10^6^ erythrocytes per second, whereas adult mice make approximately 7,000 erythrocytes per second (Palis, 2014). Erythropoiesis is characterized by the movement of erythroid lineage committed cells through three different compartments, erythroid progenitors, precursors and mature RBCs (An and Mohandas, 2011). Erythroid progenitors consist of a burst-forming unit erythroid (BFU-E) and colony-forming unit erythroid (CFU-E) (Palis, 2014) cells. These cells give rise to nucleated erythroid precursors that progress from proerythroblast, through basophilic, polychromatic and orthochromatic erythroblasts. During precursor maturation the size and RNA content of the cells decrease, hemoglobin synthesis increases, and there is progressive nuclear condensation. The orthochromatic erythroblasts expel the nucleus to form reticulocytes (An and Mohandas, 2011; Dzierzak and Philipsen, 2013; Palis, 2014). Erythroid differentiation occurs in a specific niche in the BM, the erythroblastic islands (EBIs). EBIs consist of a central macrophage surrounded by developing erythroid cells from CFU-E to enucleating erythroblasts (Bessis, 1958; Manwani and Bieker, 2008). Circulating reticulocytes and mature RBCs are the final products of the erythropoietic process.

Bone marrow is a spongy tissue that fills the cavity of most bones. BM is usually regarded as being homogeneous and sampling bone marrow from one bone, such as the femur, is thought to be representative of the whole BM “organ” (Lassailly et al., 2013). However, bones are heterogenous in their cellular embryonic origin and mode of ossification. Different bones originate from three distinct embryonic lineages. The flat bones of the skull, clavicle and cranium develop from cranial neural crest cells. The axial skeleton (the vertebral column and associated ribs) comes from paraxial mesoderm, and the appendicular skeleton (the limbs) comes from lateral plate mesoderm (Kornak and Mundlos, 2003; Provot et al., 2013). Bones have different modes of ossification; endochondral ossification is involved in the growth of long bones and intramembranous ossification occurs in cranial bones (Provot et al., 2013). In mice, the long bones and calvaria were shown to be heterogenous in bone remodeling activity, blood volume fraction, HSCs function (Lassailly et al., 2013), osteoclast activity (Everts et al., 2009), osteoblast activity (Quarto et al., 2010), hormonal and neuronal responses (Everts et al., 2009) and HSPCs hypoxic state (Lassailly et al., 2013). In humans, by 18 years of age the sites of hematopoiesis decrease in long bones and persist in the skull, sternum, ribs, vertebrae, pelvis and epiphyseal regions of femur, and humerus (Ershler and Longo, 2010). Thus, there appear to be functional differences in the cells that reside in these bones. It is not known though, if there are differences in the rate of steady state adult erythropoiesis in the different mouse bones.

The spleen is one of the main hematopoietic organs in mice during development and exhibits extramedullary hematopoiesis in physiological stress and pathological conditions. During embryonic development definitive erythropoiesis shifts from the fetal liver to the spleen, and around the time of birth, the site of hematopoiesis switches to the bone marrow from the spleen (Barminko et al., 2016). It was reported that the myelopoiesis support activity in the spleen is lost by a week after birth (Ohno et al., 1993). However, it is unclear at what age splenic erythropoietic activity is lost.

In this study, we examined steady state adult mouse BM erythroid precursors and progenitors isolated from several skeletal sites: the femur, tibia, pelvis, sternum, vertebrae, radius, humerus, frontal, and parietal bones. We compared all sites to the femur to learn whether sampling bone marrow from the femur is representative of erythropoiesis in the whole BM organ. In addition, we examined trabecular (spongy) bone and cortical (compact) bone erythroid precursors and progenitors in the femur to determine whether there are differences in erythropoiesis in the same bone. Postnatal spleen was studied to determine the age at which erythropoietic activity is lost. We provide evidence that there are steady state differences in erythroid precursors among different bones, and within the trabecular and cortical regions of the same bone. Postnatal spleen supports erythroid precursors until 5–6 weeks of age. These differences need to be taken into consideration in studies of erythropoiesis.

## Materials and Methods

### Mice

Four-month-old, male C57BL/6 mice were used for bone marrow analyses. Male mice at age of 1, 3–6, 8, and 12 weeks were used for spleen analyses. Animal housing and maintenance were in full compliance with NIH criteria for care and use of laboratory animals. The Animal Care and Use Committee of National Institute of Dental and Craniofacial Research (NIDCR), NIH, approved all the procedures employed.

### Bone Marrow and Splenocyte Collection and Preparation

Bones were cleaned of soft tissue and BM isolated using three different methods. BM from the tibia, femur, pelvis, and humerus was collected using the spin method. BM was harvested by placing the longitudinally cut bones in a perforated 0.5 ml centrifuge tube that is inserted into a 1.5 ml centrifuge tube. Following centrifugation for 30 s at 8,000 × *g* (Dobson et al., 1999), the BM is harvested from the tube. BM from the sternum and the vertebrae (Thoracic vertebrae) were isolated by crushing the bones in a αMEM medium with 2% FBS using a mortar and pestle. BM from the radius, parietal and frontal bones was collected by homogenization using a Medimachine tissue homogenizer (Becton Dickinson). Following crushing and homogenization the BM cell suspension was filtered through a 70 μm cell strainer to remove bone debris. Splenocytes were prepared by pushing the spleen through a 40 μm cell strainer with the plunger of a syringe.

### Antibodies and Flow Cytometry

Bone marrow and spleen cells were stained for flow cytometry as previously described (Mayer et al., 2017). Gating strategy for BM erythroid cells was described in the Supplementary Figure 1. Cells were stained with the following antibodies: CD45-FITC (30-F11, eBioscience), CD11b-FITC (M1/70, eBioscience), Gr-1-FITC (RB6-8C5, eBioscience), Ter119-APC (TER119, Biolegend) and CD44-PE (IM7, Biolegend), F4/80-APC (BM8, Biolegend), Gr-1-FITC (RB6-8C5, Biolegend), CD3-PE (145-2C11, Biolegend), Ly6C-PE (HK1.4, Biolegend), CD106-PE (429, Biolegend), CD45R/B220-APC (RA3.682, Biolegend). Cells were analyzed using AccuriC6 flow cytometer and data were analyzed using FlowJo software (v10.7.1).

### Colony-Forming Assay

Colony-forming assays were performed in MethoCult M3434 (Stemcell Technologies) using whole bone marrow and total spleen cells. Colonies were counted using a STEMvision automated CFU colony counter (Stemcell Technologies) on day 7.

### Blood Counts

A 50 μl of blood was collected by retro-orbital puncture at each time point. Complete blood counts were obtained with IDEXX ProCyte Dx hematology analyzer.

### Statistical Analysis

Statistical analyses were performed with Prism 8 (GraphPad Software, Inc.) one-way ANOVA followed by Tukey’s correction for different bones and unpaired *t* test. Significance is indicated by ^∗^*p* < 0.05, ^∗∗^*p* < 0.01, ^∗∗∗^*p* < 0.001, ^****^p < 0.0001.

## Results

### Steady-State Bone Marrow Erythroid Precursors and Progenitors in Different Bones

**FIGURE 1:**
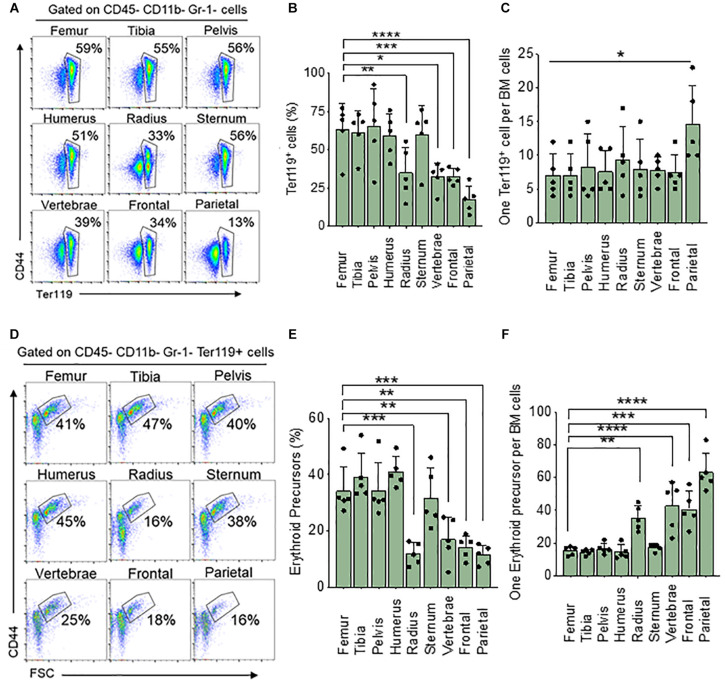
Differences in bone marrow Ter119^+^ cells and erythroid precursor from different bones. **(A)** Representative FACS plots showing expression of BM Ter119^+^ cells from different bones. The gated region shows percentage of Ter119^+^ cells within the BM. **(B)** Percentage of BM Ter119^+^ cells from different bones. **(C)** Ratio of Ter119^+^ cells to BM cells. **(D)** Representative FACS plots showing expression of BM Ter119^+^ erythroid precursors from different bones based on CD44 expression and size (FSC-forward scatter). **(E)** percentage of erythroid precursors within the Ter119^+^ cells from different bones. **(F)** Ratio of erythroid precursors to BM cells. *n* = 5 in five independent experiments. Data represented as mean ± SD; Statistical significance was assessed using one-way ANOVA followed by Tukey’s multiple comparison test. Significance was shown only in relation to the femur. *p < 0.05, **p < 0.01, ***p < 0.001, ****p < 0.0001.

We wanted to see if steady state levels of erythroid cells in the femoral BM are representative of BM in a variety of skeletal bones. To do this we analyzed BM from the femur, tibia, pelvis, humerus, radius, sternum, vertebrae, frontal and parietal bones and measured the total percentage of all erythroid (nucleated and non-nucleated) cells using the Ter119 surface marker and compared all the results to the femur. We found a significantly lower percentage of Ter119^+^ cells in BM from the radius (33%), vertebrae (39%), frontal (34%), and parietal (13%) bones than that of the femur (59%) (Figures 1A,B). The total number of live BM cells isolated from different bones are different (Table 1). We calculated the absolute number of Ter119^+^ cells in the femur and found that it is significantly different from all other bones with the exception of the tibia and pelvis (Supplementary Figure 2A). Since there are differences in the number of BM cells between bones, we also looked at the ratio of Ter119^+^ cells to BM cells. Ratio analysis shows that only parietal bone has significantly lower Ter119^+^ to BM cell ratio (1:15) compared to femur (1:7) (Figure 1C).

**TABLE 1:** BM yield from different bones.

Different bones	Cell number (×10^6^) using different methods	Cell number (×10^6^) using mortar and pestle
Femur	29.1 ± 6.4	29.1 ± 4.7
Tibia	19.4 ± 6.0	19.3 ± 6.4
Sacrum	22.0 ± 7.0	22.9 ± 5.1
Humerus	11.1 ± 2.0	14.0 ± 3.2
Parietal	0.8 ± 0.5	1.3 ± 0.3
Frontal	1.9 ± 0.4	2.2 ± 1.4
Radius	1.6 ± 2.3	2.5 ± 0.5
Vertebrae	8.2 ± 8.5	9.7 ± 6.5
Sternum	8.5 ± 4.8	10.4 ± 0.6
	Mean ± SD are shown (*n* = 5)	Mean ± SD are shown (*n* = 4)

*Absolute number of BM cells from different bones.*

Erythroid precursors (proerythroblasts and basophilic, polychromatic, and orthochromatic erythroblasts) are significantly decreased in the radius (16%), vertebrae (25%) frontal (18%), and parietal (16%) compared to the femur (41%) (Figures 1D,E). Absolute number of erythroid precursors are significantly lower in all the bones except for pelvis compared to femur (Supplementary Figure 2B). Ratio of erythroid precursor to BM cells show that radius (1:35), vertebrae (1:43) frontal (1:41), and parietal (1:66) bones have significantly lower ratio compared to the femur (1:15) (Figure 1F). No significant difference was observed in erythroid precursors between radius, vertebrae, frontal, and parietal BM. The difference in significance in the ratio of Ter119^+^ compared to erythroid precursors between femur and other bones could be due to the circulating red blood cells and reticulocytes which are included in the Ter119^+^ cells. We next isolated BM cells from all bones using manual crushing with mortar and pestle to see if the difference in Ter119^+^ cells and erythroid precursors between the bones is related to our different methods of isolating the bone marrow. The total number of cells isolated was similar among the different methods of isolation (Table 1). The percentage and the ratio of Ter119^+^ cells and erythroid precursors were also similar between the methods (Supplementary Figure 3), suggesting that the difference between the bones is not due to different isolation methods of BM from different bones.

**FIGURE 2:**
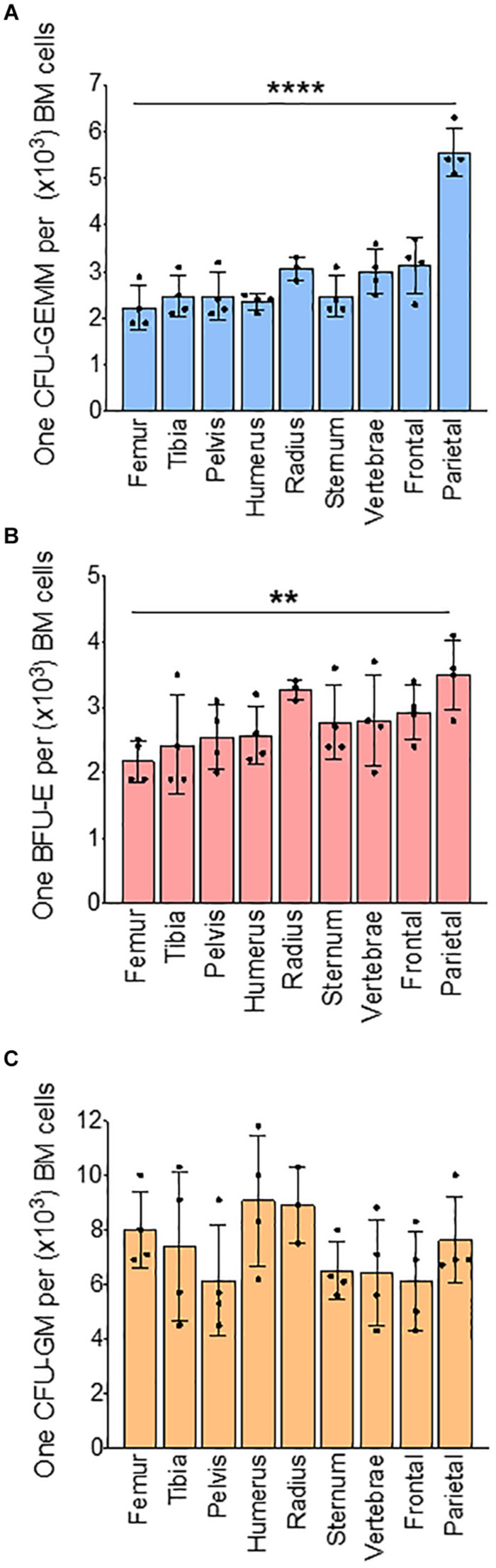
Bone marrow progenitors from different bones. Results of methylcellulose colony forming assay of whole BM from different bones. **(A)** Ratio of CFU-GEMM to BM cells **(B)** Ratio of BFU-E to BM cells. **(C)** Ratio of CFU-GM to BM cells. *n* = 4 mice in four independent experiments. Data represented as mean ± SD; Statistical significance was assessed using one-way ANOVA followed by Tukey’s multiple comparison test. Significance was shown only in relation to the femur. **p < 0.01, ****p < 0.0001.

We next analyzed BM progenitors in a methylcellulose assay using whole BM. Analysis of colonies based on the number of cells used in methylcellulose assay show that the number of CFU-GEMM colonies, representative of multipotential progenitor cells (MPPs), are significantly lower in the radius (73%), vertebrae (77%), frontal (75%), and parietal (52%) bones when compared to femur (100%) (Supplementary Figure 4B). Erythroid progenitor (BFU-E) colony numbers are significantly decreased only in the parietal BM (56%) compared to the femur (100%) (Supplementary Figure 4C). Myeloid progenitor (CFU-GM) colonies are similar in all bones (Supplementary Figure 4D). Since the total number of BM cells isolated from different bones are different, we calculated absolute colony numbers per bone. Absolute colony numbers of CFU-GEMM are significantly lower in all the bones expect pelvis compared to femur (Supplementary Figure 5A). The number of BFU-Es are significantly lower in all bones compared to the femur (Supplementary Figure 5B). CFU-GMs are significantly lower in all the bones except for the tibia and pelvis compared to the femur (Supplementary Figure 5C). Ratio analysis show that CFU-GEMM to BM cells ratio was similar between the femur (1:2,200) and other bones except for lower in parietal bone (1:5,800) (Figure 2A). Ratio of BFU-E to BM cells was also significantly lower in parietal bone (1:3,500) compared to femur (1:2,100) (Figure 2B). Ratio of CFU-GM to BM cells was not significantly different between femur and other bones (Figure 2C). Taken together, these results suggest that at steady state BM erythroid precursors of the femur are representative of the tibia, pelvis, humerus, and sternum, and erythroid progenitors are similar in BM of all the bones except for the parietal BM.

### Differences in Steady-State Erythroid Precursors and Progenitors in Bone Marrow From Trabecular and Cortical Regions of the Femur

**FIGURE 3:**
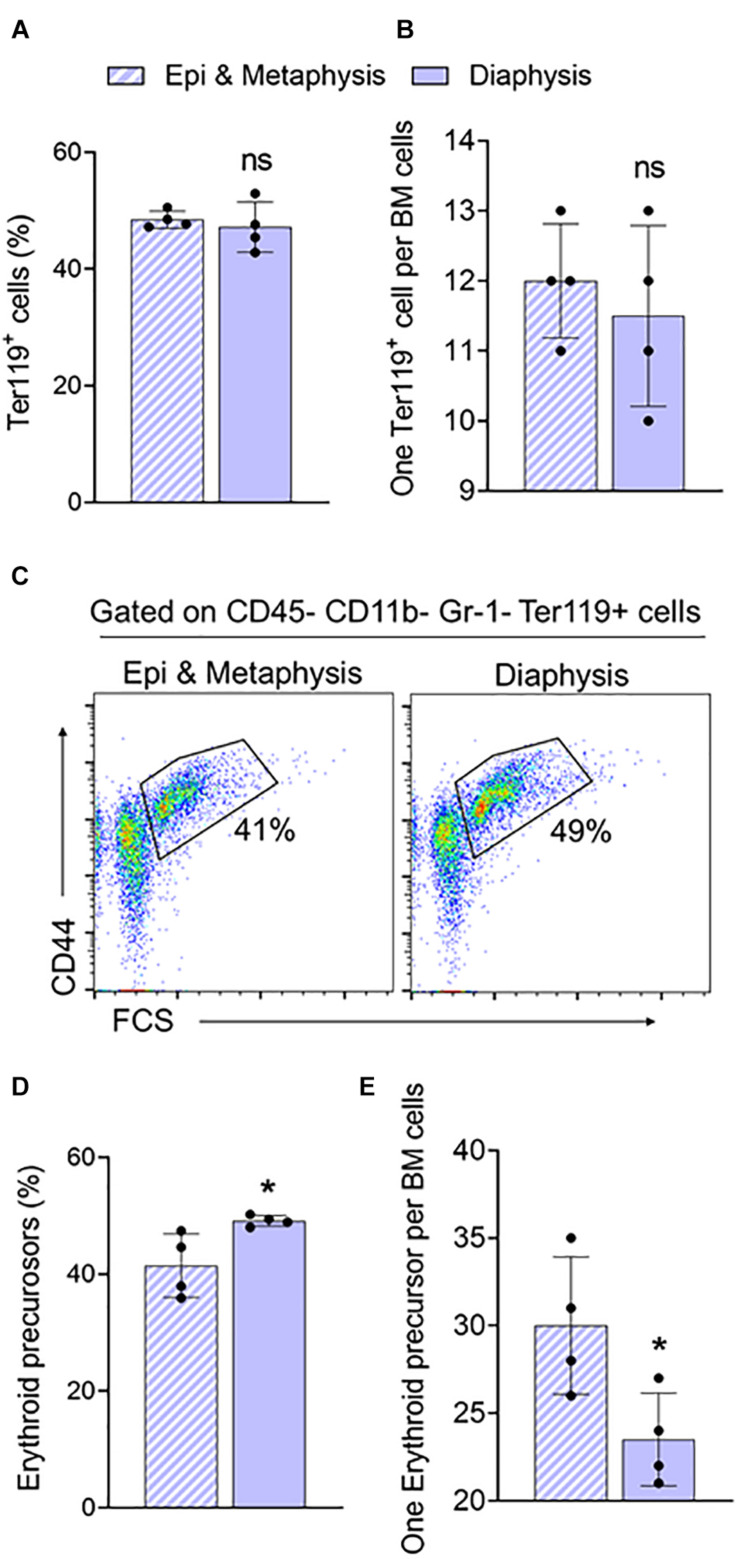
Femur epiphysis and metaphysis have lower erythroid precursors compared to the diaphysis. **(A)** Percentage of Ter119^+^ cells in epi and metaphysis, and diaphysis of the femur. **(B)** Ratio of Ter119^+^ cells to BM cells. **(C)** Representative FACS plots showing expression of BM Ter119^+^ erythroid precursors from epi and metaphysis and diaphysis based on CD44 expression and size (FSC-forward scatter). The gated region shows percentage of erythroid precursor cells. **(D)** Erythroid precursors within Ter119^+^ cells from epi and metaphysis and diaphysis. **(E)** Ratio of erythroid precursors to BM cells. *n* = 4 mice in two independent experiments. Data represented as mean ± SD; Statistical significance was assessed using an unpaired *t* test. *p < 0.05.

**FIGURE 4:**
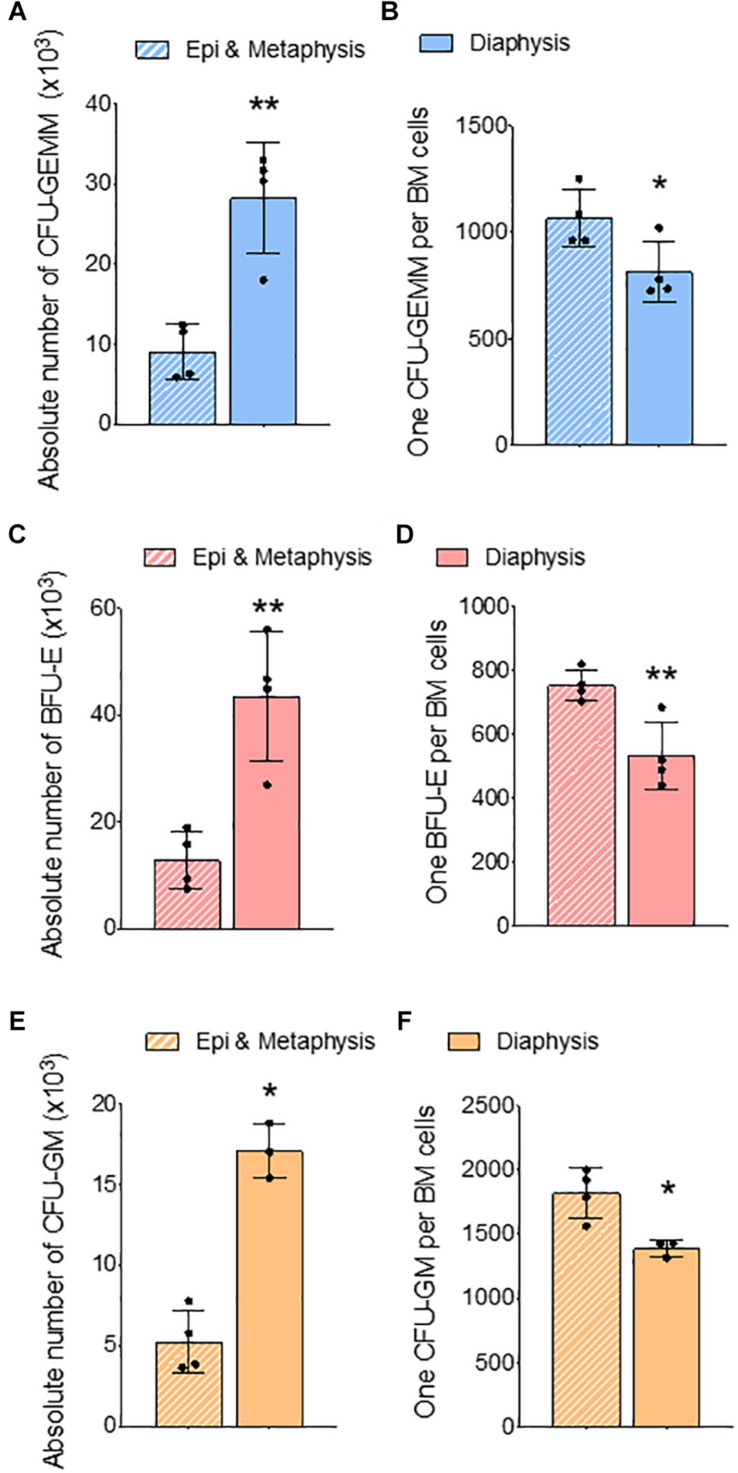
Femur epiphysis and metaphysis have lower erythroid progenitors compared to the diaphysis. Methylcellulose colony forming assay of whole BM from epi and metaphysis and diaphysis of femur. **(A)** Absolute number of CFU-GEMM in epi and metaphysis and diaphysis. **(B)** Ratio of CFU-GEMM to BM cells. **(C)** Absolute number of BFU-E in epiphysis and metaphysis and diaphysis. **(D)** Ratio of BFU-E to BM cells. **(E)** Absolute number of CFU-GM in epiphysis and metaphysis and diaphysis. **(F)** Ratio of CFU-GM to BM cells. *n* = 4 mice in two independent experiments. Data represented as mean ± SD; Statistical significance was assessed using an unpaired *t* test. *p < 0.05, **p < 0.01.

In the long bones, the epiphysis and metaphysis consist of trabecular bone, whereas the diaphysis consists of cortical bone (Gurkan and Akkus, 2008). HSCs isolated from trabecular BM have higher regenerative and self-renewal capacity than cells from cortical BM (Guezguez et al., 2013). We compared erythroid precursors and progenitors in BM isolated from epi & metaphysis, and diaphysis of the femur to see if the bone composition influences erythropoiesis. The percentages and the ratio of Ter119^+^ cells were similar in epi & metaphysis, and diaphysis (Figures 3A,B). However, erythroid precursors were significantly more abundant in the diaphysis than in epi- and metaphysis (Figures 3C,D,E). CFU-GEMM (Figures 4A,B), BFU-E (Figures 4C,D) and CFU-GM (Figures 4E,F) absolute colony numbers and their ratio to total BM cells were significantly lower in the epi & metaphysis, compared to diaphysis. These results suggest that the erythropoiesis is lower in the trabecular bone compared to cortical bone in the femur.

### Postnatal Spleen Erythropoietic Support

**FIGURE 5:**
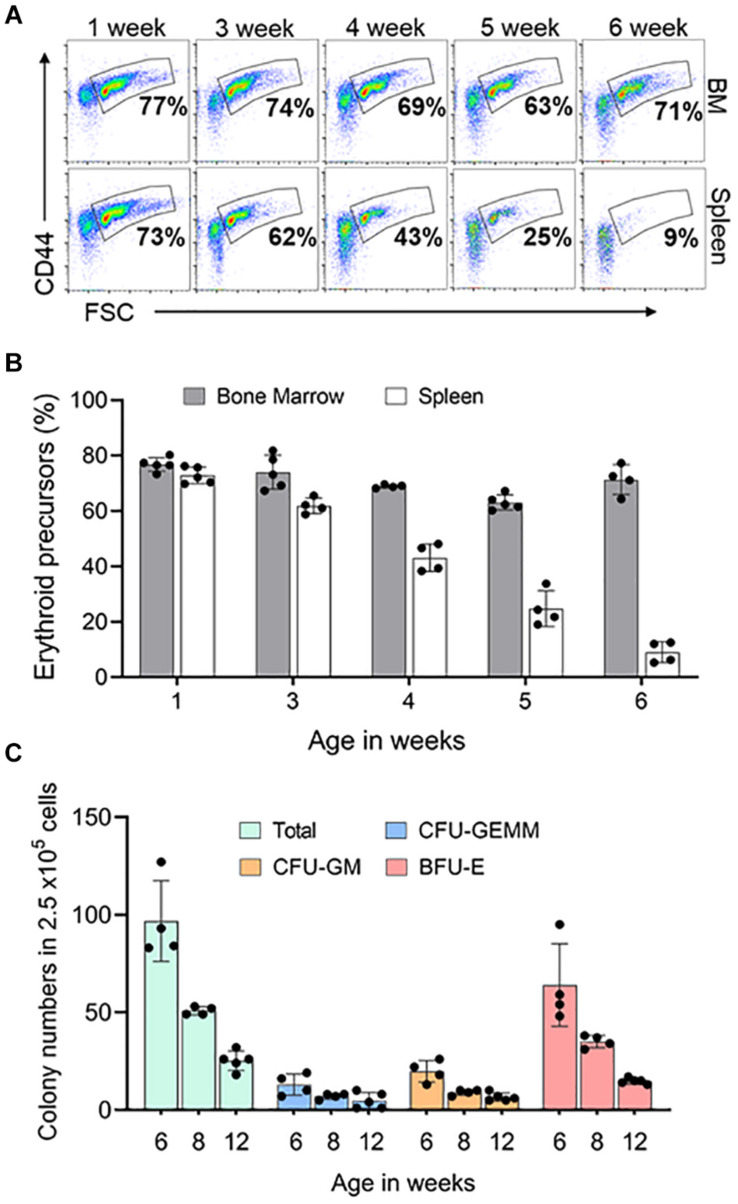
Postnatal spleen erythroid precursors and progenitors. **(A)** Representative FACS plots showing expression of Ter119^+^ erythroid precursors from bone marrow and spleen based on CD44 expression and size (FSC-forward scatter). The gated region shows the percentage of erythroid precursor cells. **(B)** Quantification of erythroid precursors within Ter119^+^ cells over time **(C)** Methylcellulose colony forming assay of the spleen at three time points. Data represented as mean ± SD; *n* = 4–5 mice per group in two independent experiments.

In mouse, after birth both BM and spleen are hematopoietic organs (Wolber et al., 2002). To learn when the spleen stops supporting erythropoiesis, we looked at the erythroid precursors and progenitors in the spleen and BM of mice at different ages. Erythroid precursors in the postnatal BM did not change significantly from 1 to 6 weeks. Splenic erythroid precursors began to decrease when the animals were 3 weeks old and completely disappeared by 6 weeks of age (Figures 5A,B). We next looked at the erythroid progenitors in spleen from 6 weeks of age to see if they reached adult levels. We compared 6- and 8-weeks progenitor levels to 3 months old spleen. Total number of spleen progenitors and BFU-E are four-fold and two-fold higher in 6 weeks and 8 weeks, respectively, compared to 3 months old. CFU-GEMM and CFU-GM are 2.6-fold and 1.4-fold higher in 6 weeks and 8 weeks, respectively, compared to 3 months old spleen (Figure 5C).

**FIGURE 6:**
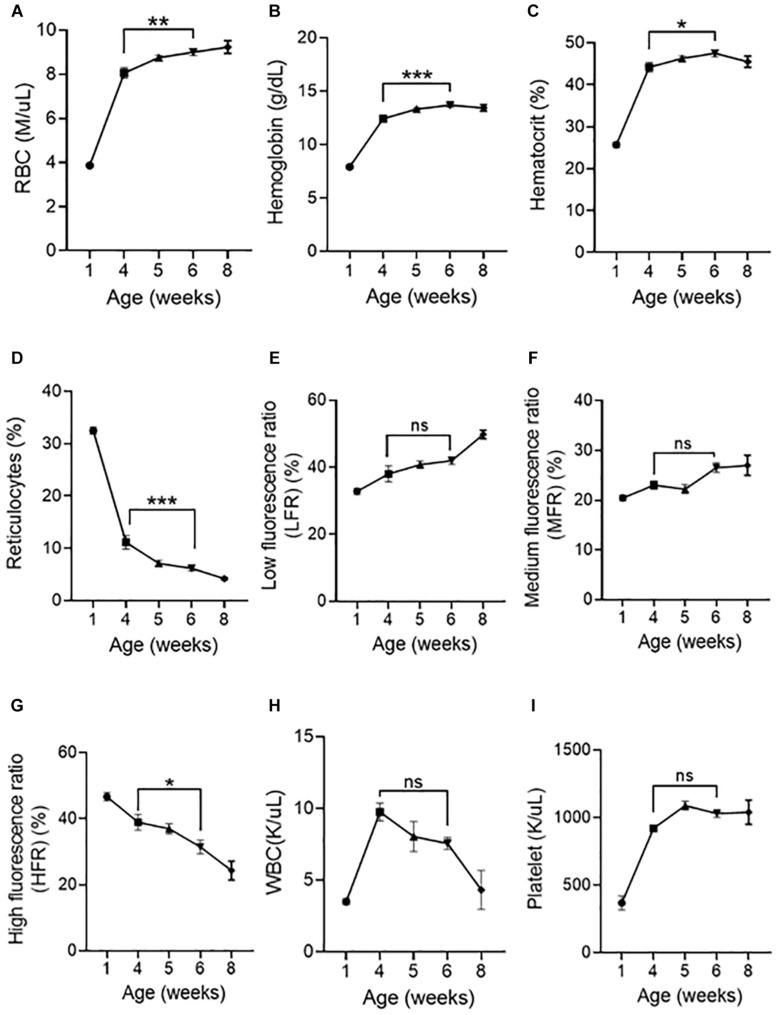
Postnatal peripheral blood analysis at different age of mice. Indices of erythroid cells at 1, 4, 5, 6, and 8 weeks of age are shown **(A)** RBC counts, **(B)** Hemoglobin, **(C)** Hematocrit, **(D)** Reticulocytes, **(E)** Low fluorescence ratio (LFR), **(F)** Medium fluorescence ratio (MFR), **(G)** High fluorescence ratio (HFR), **(H)** WBC **(I)** Platelet. Data represented as mean ± SD; *n* = 4–8 mice per group. Statistical significance was assessed using one-way ANOVA followed by Tukey’s multiple comparison test. Significance was shown between 4 and 6 weeks of age. *p < 0.05, **p < 0.01, ***p < 0.001, ns-not significant.

We next asked whether peripheral blood parameters change when the spleen loses its ability to support erythroid precursors. RBC numbers (Figure 6A), hemoglobin (Figure 6B), hematocrit (Figure 6C) significantly increased, and reticulocyte numbers (Figure 6D) have significantly decreased from 4 to 6 weeks. Analysis of reticulocyte maturity indices based on their fluorescent intensity as low fluorescence ratio (LFR), medium fluorescence ratio (MFR), and high fluorescence ratio (HFR) (Wollmann et al., 2014) show that LFR (Figure 6E) and MFR (Figure 6F) were not significantly different from 4 to 6 weeks, however, HFR (Figure 6G) significantly decreased from 4 to 6 weeks, suggesting that the number of immature reticulocytes decreases from 4 to 6 weeks. WBC (Figure 6H) and platelet numbers (Figure 6I) did not significantly change from 4 to 6 weeks.

**FIGURE 7:**
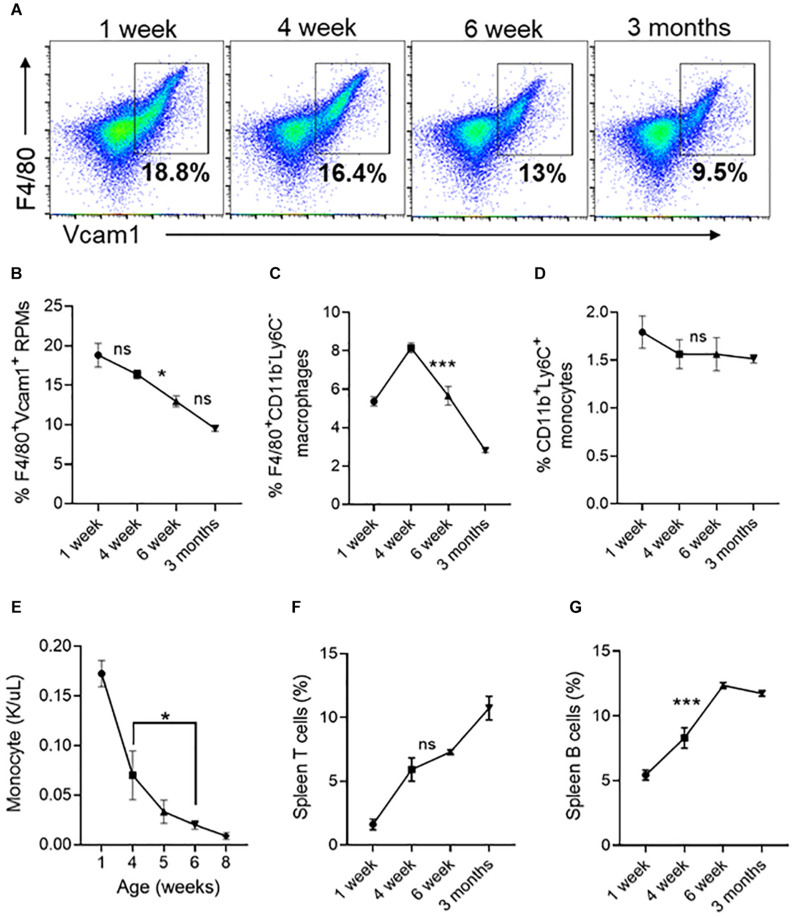
Changes in spleen red pulp macrophages, monocytes and lymphocytes at different ages. **(A)** Representative FACS plots showing expression of F4/80^+^/Vcam1^+^ spleen resident red pulp macrophages (RPM). The gated region shows the percentage of F4/80^+^/Vcam1^+^cells. **(B)** Quantification of spleen resident red pulp F4/80^+^/Vcam1^+^ macrophages at different ages of mice. **(C)** The percentage of F4/80^+^/CD11b^–^ /Ly6C^–^ macrophages. **(D)** The percentage of F4/80^–^ /CD11b^+^/Ly6C^+^ monocytes. **(E)** Monocyte concentration in peripheral blood. **(F)** Percentage of T cells. **(G)** Percentage of B cells. Data represented as mean ± SD; *n* = 4 mice per group. Statistical significance was assessed using one-way ANOVA followed by Tukey’s multiple comparison test. Significance was shown only between 4- and 6-weeks age. *p < 0.05, ***p < 0.001, ns-not significant.

We next looked at resident red pulp macrophages (RPMs) and other lineage cells in the spleen, which serves as a site for extramedullary hematopoiesis (Tavassoli and Weiss, 1973; Kurotaki et al., 2015). We found that the number of RPMs (Figures 7A,B) and F4/80^+^ macrophages (Figure 7C) are significantly decreased between 4 and 6 weeks. Between 4 and 6 weeks the percentage of monocytes in the spleen did not change (Figure 7D) but the circulating monocyte levels significantly decreased (Figure 7E). During the same period the spleen T cells showed an increasing trend (Figure 7F), and the B-cells were significantly increased by 1.5% (Figure 7G). Overall, these results suggest that the spleen contributes to erythroid precursors up to 6 weeks of age and the loss of erythroid precursor support activity coincides with a reduction in the number of red pulp macrophages.

## Discussion

In this study, we examined steady state erythroid precursor and progenitors of different mouse bones, trabecular and cortical regions of the femur, and juvenile spleen. In adult mice, we observed difference in the percentage of BM Ter119^+^ cells, erythroid precursors and MPPs of different bones compared to femur. These bones are categorized into two groups, group one, bones that have BM Ter119^+^ cells, erythroid precursors and MPPs comparable to femur are tibia, pelvis, humerus, sternum. Group two, bones that have a significantly lower percentage than femur are radius, vertebrae, frontal, and parietal bones. We also report that within femur, the epiphysis and metaphysis region (trabecular bone) have lower numbers of erythroid precursors, progenitors, MPPs and myeloid progenitors than the diaphysis (cortical bone).

The differences in erythropoiesis among the different bones described in this work were predominantly observed at the level of the erythroid precursors. This might be due to increased/decreased number or intrinsic differences among the central macrophages in the BM microenvironment in the different bones. Zhang et al. (2021) recently reported that a small subset of endothelial cells of BM sinusoids expresses the colony-stimulating factor 1 (CSF1). This endothelial cell derived CSF1 regulates the differentiation of myeloid cells (Zhang et al., 2021) suggesting that local cues by different blood vessels might control myeloid differentiation. The differences we found in erythropoiesis among the different bones could also be due to these CSF1 producing subtype of sinusoid vessels affecting the differentiation of central macrophages of the EBIs. Central macrophages of EBI were initially identified by the presence of a combination of specific adhesion molecules, such as F4/80^+^, VCAM1^+^, and CD169^+^ (Seu et al., 2017; Li et al., 2020). However, selective depletion of CD169^+^ macrophages in steady state decreased the frequency of EBI in the BM but did not result in anemia (Chow et al., 2013). Interestingly, similarly to central macrophages, osteomacs or osteal macrophages also express CD169^+^, F4/80^+^, VCAM1^+^. Osteomacs support osteoblasts in the maintenance of bone homeostasis and maintain HSCs in the BM (Chang et al., 2008; Batoon et al., 2019). These findings strongly suggest that it is unlikely that all F4/80^+^ VCAM1^+^ CD169^+^ macrophages are EBI central macrophages. Recently Li et al. (2019) showed that in addition to the known adhesion molecules described above the expression of Epo-receptors (Epo-R) is also necessary to define a macrophage to be central macrophage of EBI. Interestingly, osteoclast (bone removing cells) also express Epo-R just as central macrophages. Epo stimulates monocytic lineage cells by increasing BM monocytes, macrophages, preosteoclasts, and mature osteoclasts. Osteoclasts appear to be required for the Epo induced erythropoietic response and inhibition of osteoclasts blunts the Epo response (Singbrant et al., 2011; Hiram-Bab et al., 2015). Osteoclasts exhibit bone site specific differences as osteoclastic activity is higher in the long bones compared to the calvaria (Everts et al., 1999, 2009; Zenger et al., 2010). Given the differences in the osteoclast in different bones, and the similarity of osteoclasts to central macrophages we speculate that central macrophages might also display bone site specific differences. Further studies are needed to support (or defy) this hypothesis. The expression of Epo-R in both central macrophage and osteoclast may explain why Epo can simultaneously regulate erythropoiesis and bone homeostasis.

To date, a limited number of studies have focused on differences in the numbers and functions of HSCs in different regions inside bones (Guezguez et al., 2013; Lassailly et al., 2013). Our work shows that different regions within femur show differences in erythropoiesis. We show that erythroid progenitors and precursors in the Epi and metaphysis are significantly lower than that of diaphysis regions of the femur. These differences could be due the specific type of vasculature in these regions, which might have influenced the formation of the central macrophage of the EBI. The type of stromal cell population associated with vasculature creates the specific environment where HSCs and HSPCs are maintained. Metaphysis has type H vessels which express high levels of CD31 and endomucin (EMCN) described as CD31^*hi*^ EMCN^*hi*^. Diaphysis has type L vessels which express lower levels of CD31 and endomucin described as CD31^*lo*^ EMCN^*lo*^ (Kusumbe et al., 2014; Ramasamy et al., 2014, 2016). The CSF1 sinusoids which support myelopoiesis described by Zhang et al. (2021) are a subset of type L vessels in the diaphysis. The higher number of CFU-GM colonies seen in the diaphysis might be due to the higher number of sinusoids in the diaphysis than in the epi- and metaphysis (Zhang et al., 2021). The CSF1 sinusoids might affect the formation of the EBIs, as well as their migration toward the sinusoids as the erythroid differentiation progresses from proerythroblast to orthochromatic erythroblasts on the EBIs (Yokoyama et al., 2003). In addition, the type of vessels in a region influences the degree of hypoxia present in different regions of the bone. Diaphysis lacks a direct arterial supply and the arteries exclusively connect to type H vessels in the metaphysis which makes the diaphysis highly hypoxic. The hypoxic environment maintaining HSCs dormancy and a low metabolic state which results in a large number of HSCs in the diaphysis compared to the metaphysis (Kusumbe et al., 2014; Ramasamy et al., 2014, 2016). These studies indicate that local cues produced by distinct blood vessels might be responsible for the differentiation of specific blood cells. This fits well with our finding that erythropoiesis seem to be different within different sites of the femur, possibly due to differences in the number of HSCs, HSPCs, and EBIs in different regions. In humans, unlike in the mouse, by the age of 18 active hematopoiesis occurs only in the vertebrae, ribs, sternum, skull, pelvis, and epiphyseal regions of humerus and femur (Ershler and Longo, 2010) suggesting a species difference in the sites of erythropoiesis.

We also demonstrate that the spleen supports erythroid precursors until 5–6 weeks of age, while the residual erythroid progenitor support capacity seems to reach adult levels by 3 months of age. The role of the spleen in supporting erythropoiesis during the postnatal period has received little attention. While this paper was being revised Chen et al. (2021) published a paper on spleen erythropoiesis in developing mice which supports our results. We have shown that in the spleen a significant decrease in erythroid precursors are seen at about 4 weeks of age. This is followed by a gradual disappearance of erythroid precursors by the end of the 6th week. This event coincides with a significant down-regulation of the total number of macrophages and the RPMs during this period. This gradual loss of precursors and RPMs parallels the appearance of well-defined white pulp, red pulp and marginal zone macrophages in the spleen (A-Gonzalez, and Castrillo, 2018). This observation suggests that the compartmentalization might play a role in the loss of erythroid precursor support. In our study the erythroid progenitors, the BFU-Es, were significantly decreased from 6 to 8 weeks and 8 weeks to 3 months old spleen. This is in contrast to the report of Wolber et al. (2002) who found no significant change in the number of BFU-Es in the spleen between 8 days and 8 weeks in mice. The difference in precursor and progenitor support suggests that the niches that support progenitors vs. precursors in the spleen are different, even though both progenitor and precursors are found in the red pulp (Inra et al., 2015). In addition, this could also be due to the differences in the cytokine environment such as the levels of SCF and CXCL12 secreted by endothelial perivascular stromal cells in the red pulp. In adult mice, stress erythropoiesis increased the number of *Scf*-expressing endothelial cells and *Scf* and *Cxcl12* expressing perivascular stromal cells in the red pulp. Conditional deletion of *Scf*-expressing endothelial cells, and *Scf* and *Cxcl12* expressing perivascular stromal cells were shown to reduce spleen erythropoiesis without effecting BM erythropoiesis (Inra et al., 2015). Splenectomy of neonatal pups did not affect their ability to grow and thrive, or alter the progenitor numbers in the BM, but the number of erythroid progenitors was increased in the liver (Wolber et al., 2002). This is similar to stress erythropoiesis induced in adult splenectomized mice, where the BM did not expand the number of progenitors to compensate for the loss of the spleen. Instead, progenitors expanded in the liver of the splenectomized mice (Lenox et al., 2009). The stress erythropoiesis in adults recapitulates the postnatal spleen erythropoiesis suggesting that endothelial and stromal cell secretion of cytokines might also be modulated during postnatal time, which requires further study. These studies suggest that in times of need (e.g., during development of bone or when stress erythropoiesis is required) the BM needs help from organs such as the spleen or the liver.

In conclusion, our work shows that steady state erythropoiesis in the mouse femur may reflect RBC production in the tibia, pelvis, humerus and sternum, however, the differences need to be considered when analyzing other bones. Furthermore, studies of erythropoiesis in mice younger than 5–6 weeks of age should consider the contribution of the spleen to RBC production early in the life and analyzed when using mice less than 6 weeks old.

## Data Availability Statement

The original contributions presented in the study are included in the article/Supplementary Material, further inquiries can be directed to the corresponding authors.

## Ethics Statement

The animal study was reviewed and approved by the Animal Care and Use Committee of National Institute of Dental and Craniofacial Research (NIDCR), NIH.

## Author Contributions

VM led the conception and design of the experiments, collected and interpreted data, and wrote the manuscript. IS collected colony forming assay data. EM interpreted the results, prepared Figures, and edited the manuscript. All authors contributed to the article and approved the submitted version.

## Conflict of Interest

The authors declare that the research was conducted in the absence of any commercial or financial relationships that could be construed as a potential conflict of interest.

## Supplementary Material

Supplementary Figure 1 Overall Gating Strategy for Ter119^+^ cells and erythroid precursors. **(A)** Overall cell population was gated based on size (FSC) and complexity (SSC) **(B)** Dead cells were excluded using 7AAD **(C)** CD45, Gr-1, CD11b cells were excluded and from the remaining population **(D)** Ter119^+^ cells were gated and **(E)** Erythroid precursors were gated based on CD44 and FSC **(F)** Isotype control for Ter119.

Supplementary Figure 2 Absolute number of Ter119^+^ cells and erythroid precursors in different bones. **(A)** Absolute number of Ter119^+^ cells **(B)** Absolute number of erythroid precursors. Table shows significance between the femur and other bones. *n* = 5 mice in five independent experiments. Data represented as mean ± SD; Statistical significance was assessed using one-way ANOVA followed by Tukey’s multiple comparison test.

Supplementary Figure 3 BM Ter119^+^ cells and erythroid precursor from different bones isolated using crush method. **(A)** Percentage of BM Ter119^+^ cells from different bones **(B)** Ratio of Ter119^+^ cells to BM cells **(C)** percentage of erythroid precursors within the Ter119^+^ cells from different bones. **(D)** Ratio of erythroid precursors to BM cells. *n* = 4 mice in four independent experiments. Data represented as mean ± SD; Statistical significance was assessed using one-way ANOVA followed by Tukey’s multiple comparison test. Significance was shown only in relation to the femur. **p* < 0.05, ***p* < 0.01, ****p* < 0.001, *****p* < 0.0001.

Supplementary Figure 4 Bone marrow progenitors from different bones. Results of methylcellulose colony forming assay of whole BM from different bones expressed per 25,000 cells. **(A)** Total colony numbers **(B)** CFU-GEMM **(C)** BFU-E **(D)** CFU-GM. *n* = 4 mice in four independent experiments. Data represented as mean ± SD; Statistical significance was assessed using one-way ANOVA followed by Tukey’s multiple comparison test. Significance was shown only in relation to the femur. ***p* < 0.01, *****p* < 0.0001.

Supplementary Figure 5 Absolute number of progenitors in different bones. **(A)** Absolute number of CFU-GEMM. **(B)** Absolute number of BFU-E. **(C)** Absolute number of CFU-GM. Table shows significance between femur and different bones. *n* = 4 mice in four independent experiments. Data represented as mean ± SD; Statistical significance was assessed using one-way ANOVA followed by Tukey’s multiple comparison test.
