# Supplementary figures and images for: Differences in Steady-State Erythropoiesis in Different Mouse Bones and Postnatal Spleen
Source: Front Cell Dev Biol. 2021 May 13;9:646646. doi: 10.3389/fcell.2021.646646 (PMC8155546; doi:10.3389/fcell.2021.646646)

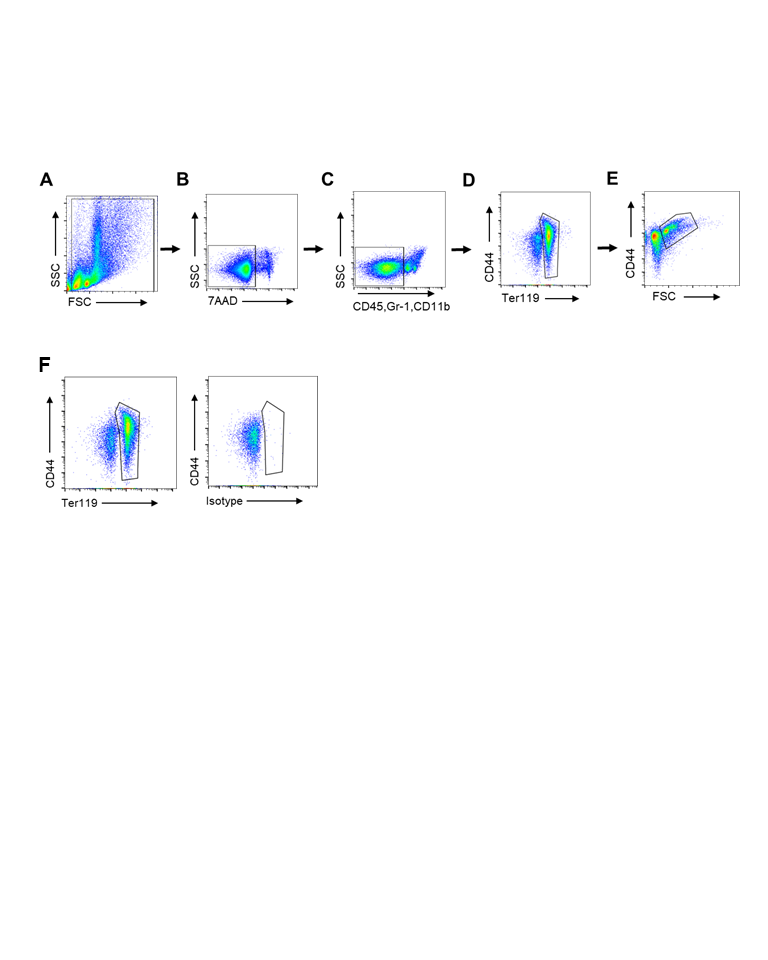

Supplement: Supplementary Figure 1 — Overall Gating Strategy for Ter119+ cells and erythroid precursors. (A) Overall cell population was gated based on size (FSC) and complexity (SSC) (B) Dead cells were excluded using 7AAD (C) CD45, Gr-1, CD11b cells were excluded and from the remaining population (D) Ter119+ cells were gated and (E) Erythroid precursors were gated based on CD44 and FSC (F) Isotype control for Ter119. [file Image_1.TIF]

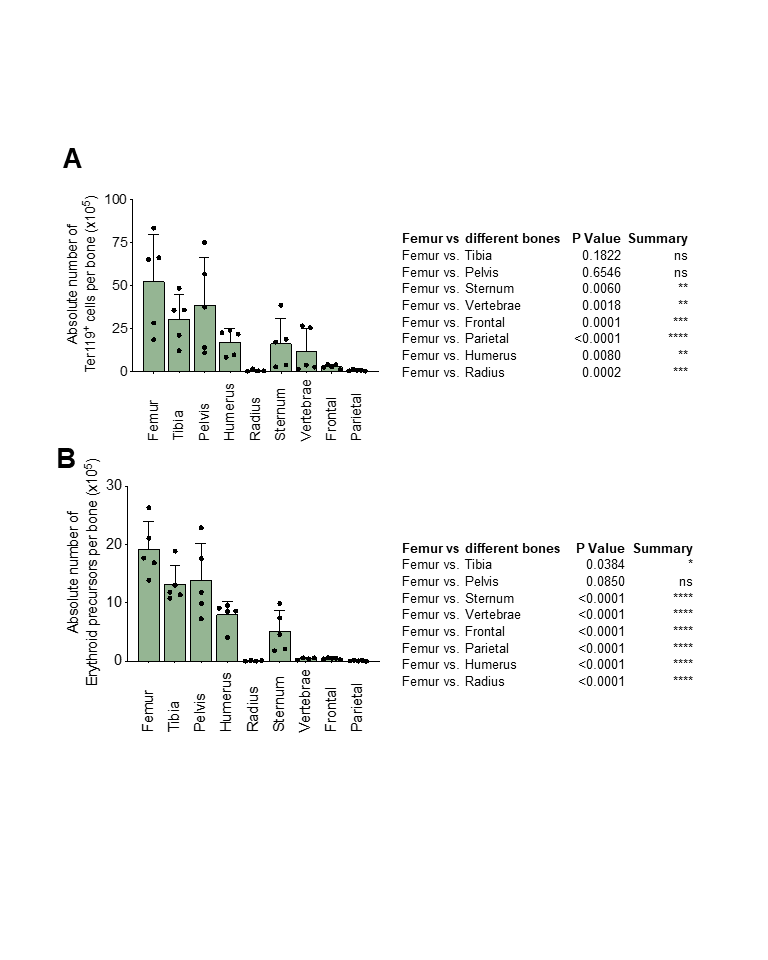

Supplement: Supplementary Figure 2 — Absolute number of Ter119+ cells and erythroid precursors in different bones. (A) Absolute number of Ter119+ cells (B) Absolute number of erythroid precursors. Table shows significance between the femur and other bones. n = 5 mice in five independent experiments. Data represented as mean ± SD; Statistical significance was assessed using one-way ANOVA followed by Tukey’s multiple comparison test. [file Image_2.TIF]

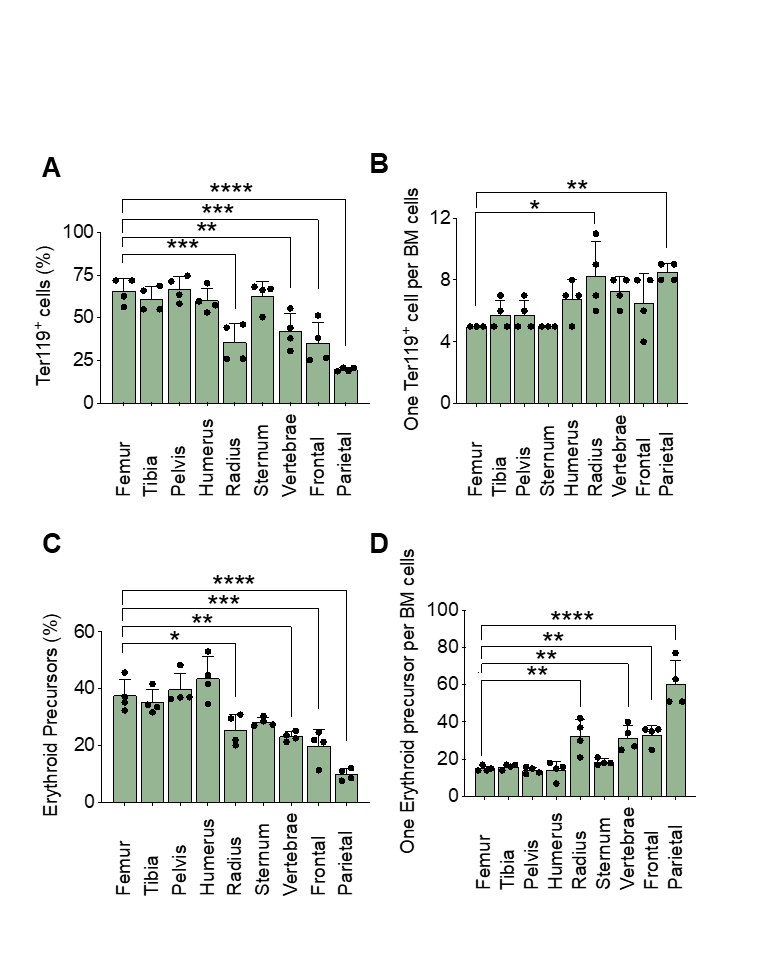

Supplement: Supplementary Figure 3 — BM Ter119+ cells and erythroid precursor from different bones isolated using crush method. (A) Percentage of BM Ter119+ cells from different bones (B) Ratio of Ter119+ cells to BM cells (C) percentage of erythroid precursors within the Ter119+ cells from different bones. (D) Ratio of erythroid precursors to BM cells. n = 4 mice in four independent experiments. Data represented as mean ± SD; Statistical significance was assessed using one-way ANOVA followed by Tukey’s multiple comparison test. Significance was shown only in relation to the femur. *p < 0.05, **p < 0.01, ***p < 0.001, ****p < 0.0001. [file Image_3.TIF]

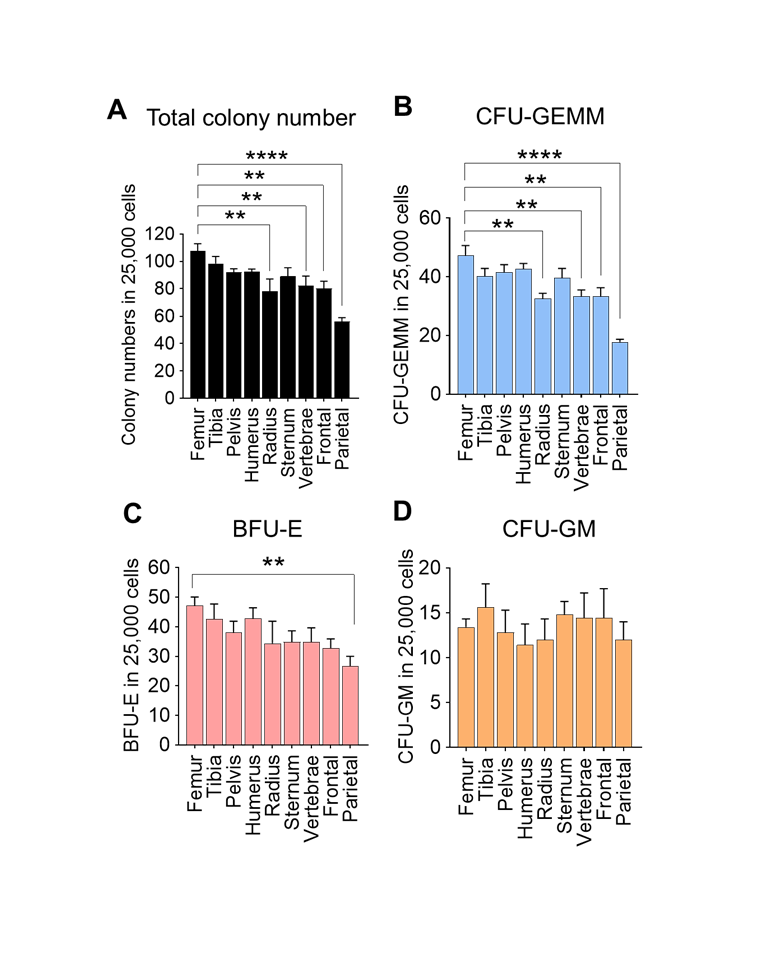

Supplement: Supplementary Figure 4 — Bone marrow progenitors from different bones. Results of methylcellulose colony forming assay of whole BM from different bones expressed per 25,000 cells. (A) Total colony numbers (B) CFU-GEMM (C) BFU-E (D) CFU-GM. n = 4 mice in four independent experiments. Data represented as mean ± SD; Statistical significance was assessed using one-way ANOVA followed by Tukey’s multiple comparison test. Significance was shown only in relation to the femur. **p < 0.01, ****p < 0.0001. [file Image_4.TIF]

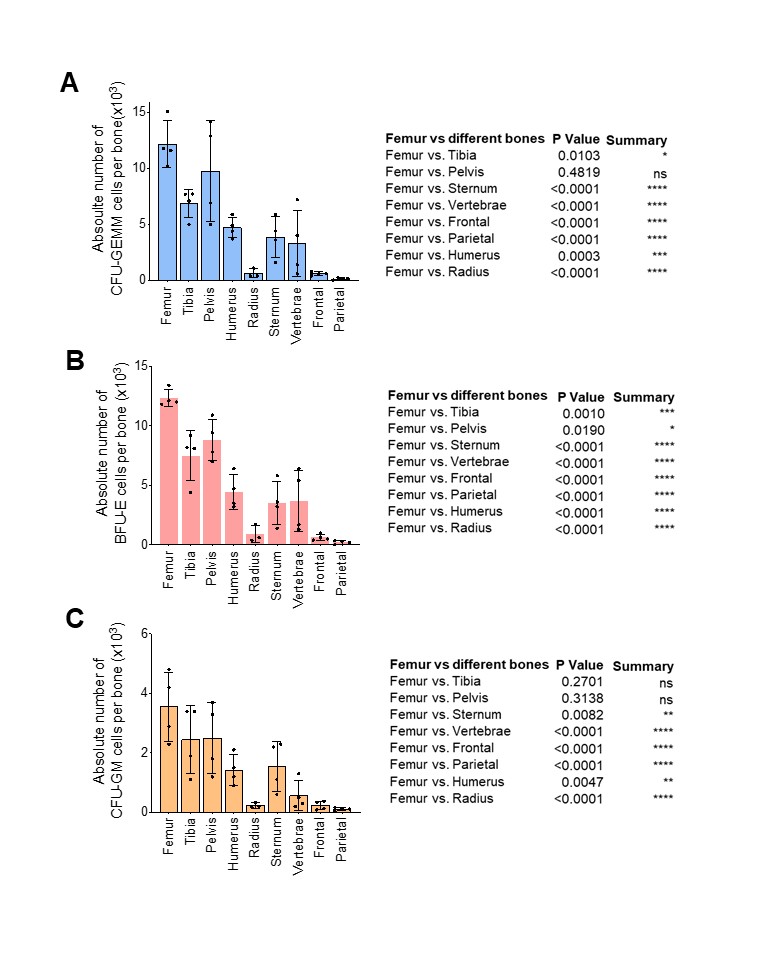

Supplement: Supplementary Figure 5 — Absolute number of progenitors in different bones. (A) Absolute number of CFU-GEMM. (B) Absolute number of BFU-E. (C) Absolute number of CFU-GM. Table shows significance between femur and different bones. n = 4 mice in four independent experiments. Data represented as mean ± SD; Statistical significance was assessed using one-way ANOVA followed by Tukey’s multiple comparison test. [file Image_5.TIF]
